# Biotensegrity of the Extracellular Matrix: Physiology, Dynamic Mechanical Balance, and Implications in Oncology and Mechanotherapy

**DOI:** 10.3389/fonc.2014.00039

**Published:** 2014-03-04

**Authors:** Irene Tadeo, Ana P. Berbegall, Luis M. Escudero, Tomás Álvaro, Rosa Noguera

**Affiliations:** ^1^Foundation INCLIVA, Hospital Clínico de Valencia, Valencia, Spain; ^2^Department of Pathology, Medical School, University of Valencia, Valencia, Spain; ^3^Instituto de Biomedicina de Sevilla, Hospital Universitario Virgen del Rocío/CSIC/Departamento de Biología Celular de la Universidad de Sevilla, Seville, Spain; ^4^Department of Pathology, Hospital de Tortosa, Verge de la Cinta, IISPV, URV, Tortosa, Spain

**Keywords:** biotensegrity, cancer, extracellular matrix, mechanotherapy, neuroblastoma

## Abstract

Cells have the capacity to convert mechanical stimuli into chemical changes. This process is based on the tensegrity principle, a mechanism of tensional integrity. To date, this principle has been demonstrated to act in physiological processes such as mechanotransduction and mechanosensing at different scales (from cell sensing through integrins to molecular mechanical interventions or even localized massage). The process involves intra- and extracellular components, including the participation of extracellular matrix (ECM) and microtubules that act as compression structures, and actin filaments which act as tension structures. The nucleus itself has its own tensegrity system which is implicated in cell proliferation, differentiation, and apoptosis. Despite present advances, only the tip of the iceberg has so far been uncovered regarding the role of ECM compounds in influencing biotensegrity in pathological processes. Groups of cells, together with the surrounding ground substance, are subject to different and specific forces that certainly influence biological processes. In this paper, we review the current knowledge on the role of ECM elements in determining biotensegrity in malignant processes and describe their implication in therapeutic response, resistance to chemo- and radiotherapy, and subsequent tumor progression. Original data based on the study of neuroblastic tumors will be provided.

## Introduction

The study of spatial and temporal responses to mechanical forces of tissue structures of biological organisms is a growing field in health sciences. Such responses can be modified by mechanotherapeutic interventions, ranging from the molecular level to whole body systems, and involving a broad spectrum of target molecules belonging to the microenvironment. In order to carry out mechanotherapy effectively, we should consider the stabilizing elements of tension and compression, or biotensegrity systems, existing at all structural levels in the body. Tensegrity is an architectural principle put forth by Buckminster Fuller in the 1960s (1, 2). According to the tensegrity principle, structures or tensegrity systems are stabilized by continuous tension with discontinuous compression (3). Coming under the term biotensegrity, the tensegrity principle applies to essentially all detectable scales in the body, from the musculoskeletal system to proteins or DNA (4, 5).

In this review, we highlight the current challenges and on-going issues for dissecting the mechanisms of tumor extracellular matrix (ECM) biotensegrity and discuss how these concepts may be translated into treatment and prognosis of cancer. To illustrate the biotensegrity principle, we present some preliminary results on the mathematical integration of multimodal data, combining imaging and non-imaging tumor tissue data, acquired in the context of neuroblastoma (NB) studies, suggesting testable hypotheses for making prognostic predictions and therapeutic response related with this principle.

## Cell and Tissue Biotensegral Physiology

Several studies have demonstrated that cells can function as independent pre-stressed tensegrity structures through their cytoskeleton architecture. Ingber defined the pre-stressed tensegrity model as a structural support on biological systems. It is constituted by a number of continuous elements of tension and a number of discontinuous elements resistant to compression providing a stabilized structure (6–14). As a tensegrity network, a single cell presents such continuous tension (mediated by cytoskeleton elements such as microfilaments and intermediate filaments) and local discontinuous compression (mediated by ECM and other cytoskeleton elements such as microtubules). The individual pre-stressed cells are poised and ready to receive mechanical signals and convert them into biochemical changes (15). Therefore, cell membrane, nucleus, and all the organelles are hard-wired by the cytoskeletal scaffold. When the mechanical cue is received, sensed mainly by focal adhesion complexes induced by integrins, the signal modifies the cytoskeletal scaffold. Thus, the local mechanical signal is amplified and propagated through a series of force-dependent biochemical reactions, whereby intra-cellular signaling pathways become sequentially activated through mechanotransduction (16). At the molecular level, several elements resist compression, such as all structures containing alpha-helix, beta-sheet, or even DNA backbone structures, while others, such as attraction and repulsion forces of molecular, atomic, and ionic bonds (such as Van der Waals forces, covalent bonds, etc.), resist continuous tension. Many molecules display such structures and are subject to these two forces at different stages along the mechanical intra-cellular signal pathways. Among these abundant and intermingled pathways, many remain unknown. We believe that knowledge on how we could potentially interfere with these signaling pathways or cascades may provide new therapeutic targets. Nevertheless, as many molecules play multiple roles in different pathways, molecular therapy based on mechanotransduction, should be carried out on specific targets to avoid adverse effects.

The tensegrity architecture of the cytoskeleton and signal pathways is linked to the tensegrity elements of the outer and inner nuclear membrane through KASH–SUN bridges, where KASH proteins are located in the outer nuclear membrane (Nesprin-1 and Nesprin-2 link nuclei with actin filaments, Nesprin-3 interacts with intermediate filaments, and Nesprin-4 binds to microtubules) (17–19) and SUN proteins in the inner nuclear membrane (Samp1 and lamin). This connection is critical for intra-cellular force transmission in physiological homeostasis (20–22) and might ensure that chromatin organization is not perturbed when tissues experience stress, and may be fundamental for normal development (23).

The self-balanced mechanical stability of the cytoskeleton enables the macro-mechanical forces to be converted into molecular changes. Since cells are connected to each other through cell junctions, mediated mainly by cadherins, these changes not only affect the cell that receives the signal, but are also transferred to the neighboring cells. Indeed, recent biophysical studies have revealed that the size of cell–cell contacts can be regulated in response to the mechanical forces exerted on those junctions and that cells are also able to regulate the forces exerted on their junctions (24–26). It is known that cell–cell junctions are anchored to neighboring cells and focal adhesions to ECM, and all are connected to the intra-cellular cytoskeletal network, therefore the forces that cross these structures fluctuate strongly when tissue is remodeled. It is becoming apparent that these structures do not just transmit forces while maintaining tissue cohesion, but also respond to fluctuations in force by actively influencing cell morphology and behavior (27). The biological significance of this mechanotransduction is to promote coordinated cytoskeletal reorganizations that can define changes in shape across the whole tissue. The basal lamina plays a central role in this process. It provides physical support to epithelial cells, surrounds muscle cells, fat cells, and Schwann cells, and is the environment where cells and ECM bind through focal adhesions and integrins. Accordingly, the shape of tissue cells (round or flattened) and the three-dimensional structure of the tissue patterns that constitute glands, alveoli, ducts, and papillae (among others), depend on the stiffness and flexibility and on the coordinated movement of the basal lamina (28).

## Role of ECM in Biotensegrity

During the last decade, cell-matrix contacts based on the transmembrane adhesion receptors from the integrin family or focal adhesions have emerged as the major mechanosensitive structural elements that connect, collect, process, and integrate the information of the ECM. Recent proteomic studies have not only found many more components, but also have revealed that many of these elements are recruited to focal adhesions in a force-dependent manner, supporting the view that focal adhesions harbor a network of mechanosensitive processes (29). Integrins are transmembrane αβ heterodimer receptors that function as structural and functional bridges between the cytoskeleton and ECM molecules. Specifically, α8β1 or tensegrin can bind to several ECM molecules and has been shown to be associated with focal adhesion points, where it participates in the regulation of spreading, adhesion, growth, and survival in different neuronal and mesenchymal-derived cell types (30, 31).

**Figure 1:**
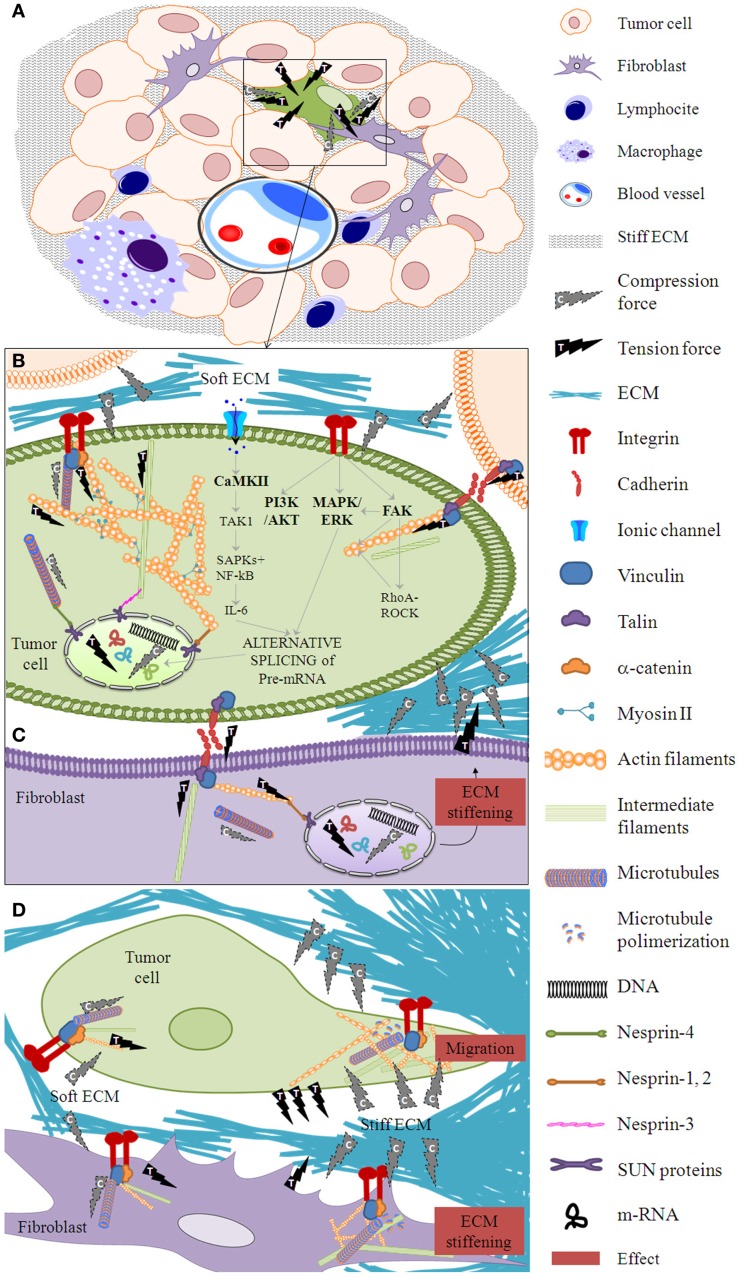
**Mechanotransduction phases**. **(A)** When forces are exerted on a tissue after proliferation of tumor and stroma cells, a cell within the cell mass (for example, the one marked in green) is subject to mechanical deformation in a phase called mechanocoupling. Following the principle of tensegrity at the tissue level, ECM elements apply compression forces to the tissue cells and tissue cells exert tension forces between themselves and to the ECM. Biotensegral elements of the boxed area are detailed in **(B–D)**. **(B)** This particular tumor cell activates a biochemical coupling phase in which the mechanical signal is transformed into intra-cellular biochemical signals through the integrin–cytoskeleton–nuclear matrix structure or stretch-activated cation channels within the cell membrane, among other mechanisms. Some essential players in mechanosensing are shown (44–47). **(C)** The biochemical signal is transmitted to neighbor cells (marked in purple) through cell–cell junctions. Following the principle of tensegrity at the cell level, the compression elements are the microtubules, whereas the tension elements are the intermediate filaments and the actin filaments. **(D)** A fibroblast neighbor cell can produce a stiff ECM which in turn enables the first cell to flatten and migrate through this stiffened ECM. The effector cell response phase arises from the biotensegrity principle: a simple cytoskeleton when the ECM compression is low switches to a complex cytoskeleton when the ECM exerts high compression on the cells and the cells exert high tension on the ECM.

Various interconnected cells bind to their microenvironment, forming a mechanical tensegral system, which implies the existence of a mechanical balance between compression (ECM) and tension (cell) forces. The ECM is made up of a mixture of ground substance [glycosaminoglycans (GAGs) – mostly hyaluronan, proteoglycans, and glycoproteins] situated in close relationship with a fibrous scaffold [reticulin (Ret F) – elastin and collagen fibers (Col F)], and supplies much of the structural support available to parenchymal cells in tissues, by adding tensile strength and flexibility (32). The ECM is a dynamic and multifunctional regulator and has its own biotensegrity with Ret F and elastin fibers acting as tensional elements, and ground substance and Col F as compression-resistance elements. This tensegral network is considered to be a solid-state regulatory system of all cell functions, responsible for changes in genes and proteins, as well as alterations in cell shape and movement (33–35). One result of cell–ECM biotensegrity is substrate rigidity, which can control nuclear function and hence cell function (36). Cells can use this substrate rigidity to exert traction forces, thus altering the ECM. Indeed, in a state of reciprocal isometric mechanical tension, a dynamic balance exists between cell traction forces and points of resistance within the ECM. This dynamic biotensegral system with its mechanotransduction phases (Figure 1) enables our cells to mechanosense, modifying their microenvironment, thus promoting ECM remodeling in homeostasis and in tissue disorders (37). Manipulation of this mechanical balance could be used to promote tissue regeneration. In fact, various studies have demonstrated that different elasticities of the ECM drive mesenchymal stem cell differentiation in a very specific way. Neurogenic, myogenic, or osteogenic differentiations are induced under identical matrix serum conditions, with variations in ECM softness, strength, and stiffness (38). Furthermore, ECM stiffness guides cell migration. It has been shown that fibroblasts prefer rigid substrates and when placed on flexible sheets of polyacrylamide, they migrate from the soft to the stiff areas (39). Under homeostatic conditions, collagen fibrils have a minimal turnover. However, this turnover is accelerated during tissue remodeling and tumor development, as evidenced by the serum levels of its degradation products (40). Studies of the ECM have revealed that the components of the tumor microenvironment are fundamental, not only for the regulation of tumor progression (41, 42), but also are essential even before the tumor appears. The stromal cells are able to transform the adjacent cells through an alteration in the homeostatic regulation of the tissue, including the control of architecture, adhesion, cell death, and proliferation (43).

## Cell and Tissue Biotensegrity in Cancer

Cancer can be understood as a disease of the developmental processes that govern how cells organize into tissues (48). The tumor microenvironment is comprised of a variety of cell types lying among a network of various ECM fibers merged within the interstitial fluid and gradients of several chemical compounds, which constantly interplay with malignant cells (34). Therefore, we can infer that the previously described biotensegral systems also exist within tumor tissue. In fact, the dynamic mechanical balance achieved through mechanosensors, cytoskeletal tensegrity, molecular biotensegral intra-cellular pathways, ECM with compressive and resistant elements, supportive cells (such as fibroblasts and multiple tumor-associated immune cells), and vascular and lymphatic vessels tensional structures, can be as important as the genetic instability of tumor cells in the pathogenesis and evolution of the malignant process (42, 49). In this regard, various studies have demonstrated the importance of cell–ECM biotensegrity in cancer (34, 48, 50). Indeed, a desmoplastic reaction is frequent in many solid tumors, such as breast, prostate, colon, or lung, in which high levels of TGF-b and PDGF are found. These growth factors are produced by the mesenchymal cells of the tumor stroma and induce immunophenotypic changes. These changes are observable by studying actin-alpha, myosin, vimentin, desmin, and the altered production of several ECM proteins, such as collagens, laminin, tenascin, ECM metalloproteinases (MMP), and MMP-inhibitors (43). Additionally, ECM stiffness modulates cancer progression; cancer cells promote stiffening of their environment, which in turn feeds back to increase malignant behaviors such as loss of tissue architecture and invasion (51). For instance, the speed of malignant cells *in vitro* is affected by the geometry of the ECM. Human glioma cells move faster through narrow channels than through wide channels or in non-stretched 2D surfaces. This is thought to be triggered by an increase in the polarity of the traction forces between cell and ECM (52). Indeed, recent publications describe that not only neoplastic ECM stiffness, but also the firmness of tumor cells play a significant role in tumor progression. The firmness of tumor cells, especially the metastatic cells, has been found to be lower than that of the normal cells of the same sample, and is caused by the loss of actin filaments and/or microtubules and the subsequent lower density of scaffold (53, 54). In this regard, it has been shown that the transformation from a benign proliferative cell into a malignant cell can be produced by a peculiar phenotypic change, known as epithelial–mesenchymal transition (55). This transformation involves breaking contact with sister cells and increasing motility, as a result of a change in the epithelial cytoskeleton, with its corresponding proprieties for a pseudomesenchymal phenotype, which enables migration, invasion, and dissemination (56). While normal cells adhere to their environment through integrins, and their body has a proper consistency, tumor cells lose that consistency and tensegrity, becoming easily deformable elements (causing pleomorphism), with high elasticity (enhancing infiltration) and with an increased degree of mobility (enabling metastasis) (57). In breast cancer, the genomic profile expressing mainly mesenchymal features is actually found in the most invasive cell lines (58). Moreover, it has been published that chronic growth stimulation, ECM remodeling, alteration of cell mechanics, and disruption of tissue architecture are non-genetic influences on cancer progression (42, 49). These ideas not only agree with basic predictions of cellular tensegrity, but also support the idea that therapy based on the manipulation of the biotensegrity principle cues should be considered as a way to revert the malignant phenotype (59, 60).

In cancer research, the hallmark which includes the physical aspects of tissue has been less investigated, but it is known that this hallmark is one of the most basic mechanisms in enhancing tumor proliferation and creating resistance to cancer treatment, among other processes (61). A previous study by our group takes in account some structural elements of ECM that have the capacity to influence physical conditions and suggests that Schwannian stroma cells are not the only important factor in the histopathologic analysis of neuroblastic tumors (50). Specifically, multi-parametric analysis of other tumor stroma components (Ret F, Col F, GAG, and immune cells) detected by classic histochemistry (HC) and immunohistochemistry (IHC) techniques and incorporated into a quantitative morphological analysis would improve the value of the International NB Pathology Classification (62). As we will show later, chemotherapy and radiotherapy are known to act on tumor cells as well as on stromal cells and ECM elements (63, 64). As a consequence, injury to the ECM can contribute directly to treatment resistance, creating niches of resistant tumor cells (64, 65). Furthermore, damage to DNA induces the production of cytokines and growth factors by stromal cells, this triggers inflammation, cell survival, and tumor progression, thus the effect of therapy on ECM may be to promote relapse or chemoresistance (66, 67). We hypothesize that studying the different elements of the ECM, as one of the main contributors to biotensegrity, through objective morphometric analysis and the creation of mathematical networks of histologic sections stained with HC and IHC, can shed light on how biotensegrity influences tumor microenvironment and could provide clues to its action mechanism.

## Evidences of ECM Biotensegrity Changes in Malignant Tissue

It is known that tumor cells alter the mechanical properties of the microenvironment in order to create favorable conditions for their proliferation and/or dissemination (68). In addition, adhesion molecules such as E-cadherin are involved in the processes of tissue differentiation and morphogenesis and play an important role in modulating the invasiveness of tumor cells in breast cancer and other epithelial tumors (69). For instance, the reciprocal communication between the stromal cells and the tissue parenchyma directs gene expression, and in prostate carcinoma and breast carcinoma, the oncogenic potentiality arises from stroma-associated fibroblasts, immune response, and the alterations of biotensegrity (49, 70). Deregulation and disorganization of the composition, structure, and stiffness of the ECM elements progressively increase interstitial fluid pressure, leading to limited penetration and dissemination of therapeutic agents within solid tumors, thus enabling the creation of niches within tissues and organs that offer sanctuary to tumors and activate therapy resistance programs (11–13, 64, 65). Tumor cells are not the only cells that change the mechanical properties of the microenvironment. Despite all the efforts of tumor cells to make ECM elements work for their survival and proliferation, tumor stromal cells, specifically, immune system cells, try to reverse the pathological condition. Indeed, two lymphoproliferative syndromes (follicular lymphoma and Hodgkin lymphoma) are good examples of the fact that a tumor can be considered as functional tissue, connected and dependent on the microenvironment, which sends and receives signals to and from the tumor tissue itself. In such syndromes, tumor microenvironment stromal cells, including immune response, determine the morphology, clinical stratification, aggressiveness, prognosis, and response to treatment of the tumor (71).

In the next two sub-sections, we describe the methods developed for the study of biotensegrity in neuroblastic tumors.

### Morphometric analysis of ECM elements – an example in NB

Accurate quantification of pathology specimens using imaging technology to analyze the variations in structural tissue that arise from interactions between tumor and stroma cells and ECM elements is providing important information. This approach would allow biotensegral patterns to be included in computational formulations for risk stratification systems and aid in designing better anti-cancer treatment strategies (29). However, the validity of the model depends on the quality of the data. This quantification depends on the staining, scanning, image analysis, and statistical evaluation. For that purpose, automatic stained sections must be digitized using microscopic preparation scanners such as Aperio Scanscope XT (Aperio technologies) or Panoramic Midi (3Dhistech) or with a photomicroscope if a scanner is not available. Different image analysis systems can be used, such as Image Pro-plus software (Media cybernetics), ImageScope (Aperio technologies), Panoramic viewer (3D Histech), free software (ImageJ of the NIH), or self-designed software to obtain customized *macros* or algorithms (informatic protocols) to detect and characterize the quantity (number of objects and area occupied), size (area, width, length), shape (aspect, roundness, perimeter ratio, fractal dimension), and orientation (angle), among other parameters, of the ECM elements of interest. All systems provide mark-up images or masks, which represent the recognized and measured element in white upon a black background. The use of tissue microarrays is advised for standardization purpose of background subtraction and color segmentation, given that these techniques tend to be dependent of the intensity of the staining and algorithms must be recalibrated with every change of intensity/ground noise/contrast staining, thus losing objectiveness. Further details regarding objective quantification of different cell and ECM elements and a flowchart of the analysis used by our group, are described elsewhere (50).

**Figure 2:**
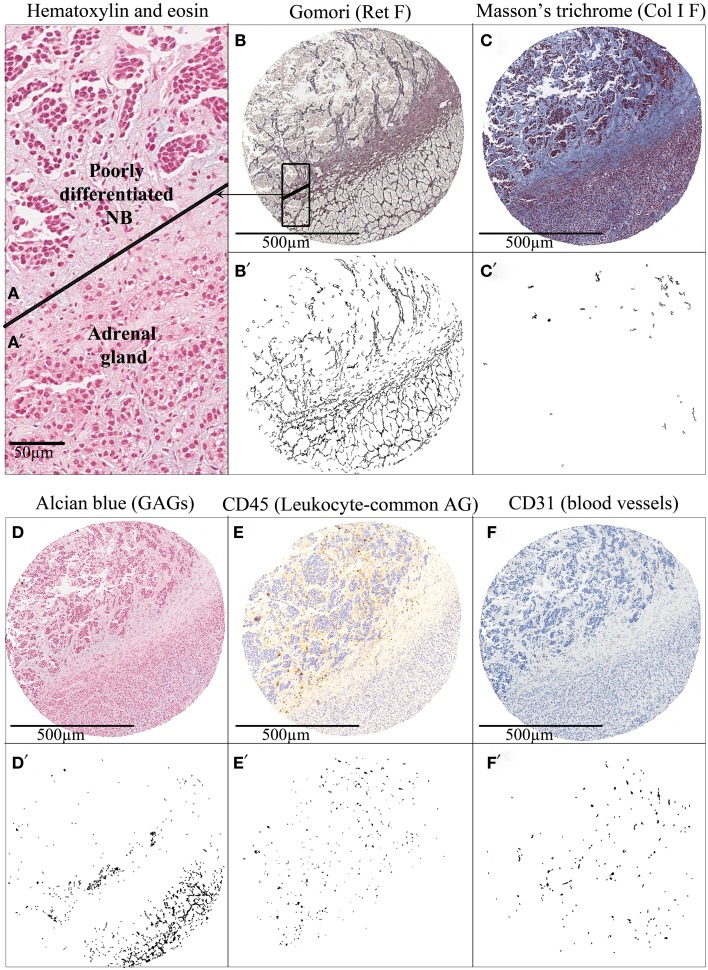
**Tissue microarray cylinders representing an adrenal gland infiltration by a poorly differentiated neuroblastoma**. Hematoxylin and eosin staining of **(A)** adrenal gland and **(A’)** tumor tissue. **(B–F)** Several histochemical and immunohistochemical stainings of extracellular matrix elements and **(B’–F’)** their corresponding mark-up images after image analysis are shown. The cylinder tissue was stained with **(B)** Gomori for Ret F, **(C)** Masson’s trichrome for Col I F, and **(D)** Alcian blue pH 2.5 for GAGs; and immunostained with **(E)** anti-CD45 for leukocyte-common antigen and **(F)** anti-CD31 for Blood vessels. Tumors tend to disrupt the topology of the ECM of the organ in which they settle, producing changes in biotensegrity. AG, antigen.

Neuroblastic cells are known to be committed in a complex interaction with the surrounding tumor microenvironment and we believe that patients with neuroblastic tumors, specifically those still subject to therapeutic failure despite current knowledge, could benefit from novel therapeutic strategies which could originate from the study of ECM biotensegrity. To investigate such new therapeutic targets, we have objectively quantified Ret F, Col I F, GAGs (Gomori, Masson’s trichrome, and Alcian blue pH 2.5 HC, respectively), blood vessels (CD31 IHC, Dako), lymph vessels (D2-40 IHC, Dako), and cell markers, including leukocyte lineage (CD45/LC IHC, Dako) and NB cells, in primary NB. A first approach to the evaluation of the role of ECM biotensegrity in neuroblastic tumors is the observation of the mark-up images of tissue microarrays cylinders comprising a mixture of tumor and normal tissue either in the primary and/or metastatic location. In the particular case presented in Figure 2, included for illustrative purpose, a clear disruption in the organization of the ECM elements can be observed when passing from the normal tissue area to the neoplastic tissue. In the tumor area, Ret F becomes disorganized, Col I F is slightly increased (although minimal), GAGs almost disappear, CD45 reactive cells accumulate, and blood vessels vary in size and characteristics.

Statistical analysis of the quantitative data of fibers, GAGs, tumor cells, and immune system markers compared with the current parameters used to predict risk of relapse (stage, age, histopathology, state of *MYCN* oncogene, state of 11q region, overall genomic profile, and ploidy) (72–74) and other genetic markers of prognostic interest in a subset of 78 primary neuroblastic tumors has already been published by our group, and highlights the interest of studying ECM in neuroblastic tumors (50). The fact that ECM elements differ depending on the characteristics of the tumors and, more interestingly, the fact that the characteristics of ECM elements are related to prognosis (relapse or overall survival) advocates on behalf of the regulatory role of ECM biotensegrity in tumor progression.

### Development of mathematical topology of ECM elements – an example in NB

**Figure 3:**
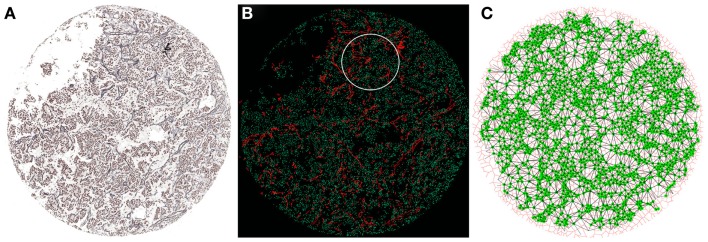
**Network construction from a NB biopsy image**. **(A)** Gomori staining to visualize Ret F and nuclei from the NB biopsy. **(B)** Combination of nuclei (green) and Ret F mark-up images obtained from **(A)**. The white circumference represents the region of interest processed in **(C)**. **(C)** Each nucleus (green) is taken as a seed to build a Voronoi diagram (red) of Voronoi cells. This allows the creation of a network of cell-to-cell contacts (black lines) that can be used to extract topological characteristics of the tissue.

The combination of multidisciplinary efforts by clinicians, biologists, pathologist, bioengineers, and biostatisticians could elucidate how ECM elements interact with tumor and stromal cells. In this respect, a new and interesting approach is to analyze biopsies by converting the tissue into a mathematical network of cell-to-cell contacts (75–78). Using graph theory concepts, these networks can provide organizational information that seems relevant in embryologic development and disease. For example, this method has been applied to the analysis of neuromuscular diseases, serving as a diagnostic tool able to quantify the severity of the pathology in a muscle biopsy (77). We propose that this technology can be adapted to the analysis of tumor biopsies. It is already possible to compare different mark-up images obtained from the analysis of several markers which have been assessed on serial thin sections with preserved histology. These overlapping images enable several markers to be considered at the same time and allow the co-location and study of the interaction between continuous tensional elements and discontinuous compression elements. In this regard, we have analyzed the relationship between different biopsy components taking the cell nuclei as a reference. The procedure is based in the identification of the cell nuclei and the calculation of their respective centroids. These centroids serve as seeds to perform a Voronoi diagram of Voronoi cells (79, 80). A new partitioned image is produced in which each nuclei is associated with its corresponding Voronoi cell. In this way, it is possible to construct a network based on the neighboring Voronoi cells. Topological approaches and the use of Voronoi cells need to be able to capture the presence and relative disposition of tissue heterogeneities derived from, for example, the luminal space of glands, blood and lymph vessels, or larger extracellular spaces. They will be reflected through different characteristics and will be taken into account for the study, testing if they can be part of the relevant features that define a specific condition. The selection of regions of interest in each biopsy will allow studying only tumor tissue areas without artifacts that could bias the study, leading to wrong conclusions. The combination of graph-related parameters with the morphometric information will enable a comprehensive analysis of the changes arising from different compression forces in relation to the different types of ECM (stiff/soft, organized/chaotic) in combination with the tumor stroma cells such as immune cell infiltrates. We have performed preliminary comparisons based on the genetic features of NB using Ret F and blood vessels (in addition to the nuclei) as the reference features for providing the biological clues. This procedure has shown some hints of discrimination regarding the organization and co-location of these elements (Figure 3). We found that some network characteristics were relevant to perform this initial separation. This suggests that diverse backgrounds can respond differently to the pathological process depending on the organization of the tumor. Following the same approach, we will use other mark-up images of the positive cells stained with the different monoclonal antibodies against the different cells of the leukocyte lineage. We hope that this combination of mathematical and statistical methods will answer the question on the relationships between ECM biotensegrity and the changes mediated by the cell infiltrate.

## Effect of Treatment on Tumor Microenvironment and Consequences

There is much evidence that the lack of total specificity of cancer therapeutic agents (chemotherapy and radiotherapy) causes collateral damage to the mechanical properties of the tumor ECM and benign stromal cells (which were previously fighting the tumor), creating resistance to therapy and favoring relapse and metastasis. This fact becomes evident while analyzing post-treatment biopsies, which contain a high degree of fibrosis and calcification. Some studies have shown that cancer therapy can sometimes damage tumor DNA and stromal cells, which results in the secretion of a spectrum of proteins, including the Wnt family members. For example, in prostate cancer, the expression of this proteins in the tumor microenvironment, regulated by lymph B cells, attenuates the effects of cytotoxic chemotherapy *in vivo*, promoting tumor cell survival and disease progression (63, 81). It has also been reported that in follicular lymphoma and diffuse large cell lymphoma, treatment with lenalidomide affects the immune synapses of intra-tumoral T lymphocytes (82). In breast cancer, treatment with doxorubicin results in an increase in fibuline-1, an ECM protein, and its binding proteins, fibronectin and laminin-1, which constitute a source of chemoresistance (83) and triggers overexpression of maspin protein, which induces the accumulation of collagen fibers, thus causing disease progression (84). A novel Toll receptor-9-dependent mechanism that initiates tumor regrowth after local radiotherapy has also been reported (85). Monoclonal antibodies against fibulin-1 are able to reverse such chemoresistance, and the inhibition of MMP seems to have a therapeutic effect (86).

**Figure 4:**
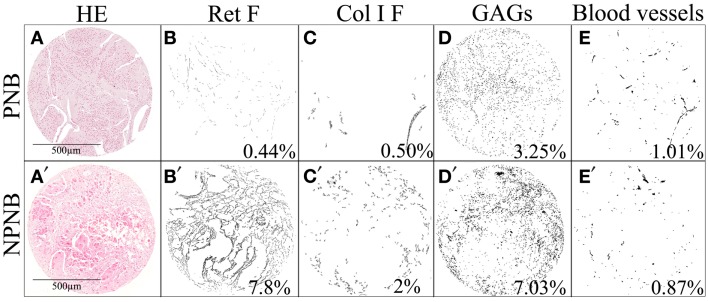
**Changes in the ECM after treatment in one neuroblastic tumor**. Several stainings (HC and IHC) of **(A–E)** a primary neuroblastoma and **(A’–E’)** its post-treatment biopsy are shown. The presented mark-up images of **(B,B’)** Ret F, **(C,C’)** Col I F, and **(D,D’)** GAGs show that these ECM elements are increased in NPNB. Regarding blood vessels **(E,E’)**, the amount of does not seem to change after treatment but the type of vessels has changed in such a way that they are smaller in NPNB. The remodeling characteristics of the ECM components, with different mechanical properties, are differentially related to prognostically significant clinical and biological features in NB. The percentages of stained area are indicated. PNB, primary neuroblastoma; NPNB, non-primary neuroblastoma (after treatment); HE, hematoxylin and eosin.

An example of the effect of treatment in NB is shown in Figure 4. When comparing a primary NB with its non-primary sample, we can appreciate that Ret F, GAGs, and Col I F accumulate in the ECM of the post-treatment sample. The amount of blood macrovasculature is slightly decreased. All these findings describe a stiffer ECM after multimodal treatment.

## Potentiality of Mechanotherapy

The changes exerted on ECM by therapeutic agents and the perspective of the epithelial–mesenchymal transition in epithelial tumors have opened the door to a new line of treatment, which considers the genetic and epigenetic mechanisms associated with resistance to chemotherapy (59, 87). There is a need to personalize therapeutics taking into account not only the features known to have prognosis impact, but also new markers, such as the mechanical stress of the tumor ECM elements. Indeed, because of its importance to the tumor, the ECM represents an “Achilles heel” that can be exploited in designing cancer therapy. The bionetwork between the ECM and tumor cells is dynamic, and for every action, such as exposure to genotoxic stress, there are reactions and consequences throughout the micro and macrosystem (65). Removal of ECM barriers will either have a direct negative effect on tumor cells or facilitate anti-tumor immune responses and drug treatment, through better intra-tumoral penetration and accessibility to target cells. In this regard, a number of experimental approaches are aimed toward the transient degradation or down regulation of ECM proteins using injection of ECM-degrading enzymes into the tumor or their intra-tumoral expression after viral- or stem cell-based gene transfer (65). Other approaches attempt to indirectly decrease tumor-associated ECM by killing tumor stromal cells that produce ECM proteins (64) or aim at enhancing the host immune response (88). The potentiality of therapeutic agents to modify ECM can be turned around in such a way that new chemicals can be applied to modify a given ECM stiffness or composition into one shown to trigger a better prognosis.

## Concluding Remarks

Biotensegrity is the structural principle of mechanotherapy. Cells are linked both to each other and to the ECM forming a mechanical biotensegral system in homeostasis. Cell–cell junctions are anchored to neighboring cells and focal adhesions to ECM, allowing forces to cross via intra-cellular cytoskeletal and nuclear networks. These structures fluctuate, and the multiple responses appear to strongly affect tissue remodeling and cell transformation. As described, normal organs tissue, primary NB, and post-treatment NB have a different amount and topography of biotensegral ECM elements. Conventional approaches have traditionally focused on the neoplastic cell. Moreover, an arsenal of mechanotherapeutic approaches to enhance the efficacy of more classical cancer therapeutics and overcome treatment resistance has already been discovered. To achieve more effective personalized strategies, further studies should consider to improve the definition of the interactions between tumor and stromal cells and the ECM elements and vascular constituents of the tumor, as well as their influence on treatment. We propose that integrating the tumor topo-functional networks of the ECM elements with the clinical, histopathology, and genetic information could provide new information about the impact of biotensegrity on patient care. Understanding the mechanical properties of tumor ECM components, related to variations in quantity, degree of interference, and types of organization, is key to defining new potential mechanotherapeutic targets and agents.

## Conflict of Interest Statement

The authors declare that the research was conducted in the absence of any commercial or financial relationships that could be construed as a potential conflict of interest.
